# Effect of protocatechuic acid-layered double hydroxide nanoparticles on diethylnitrosamine/phenobarbital-induced hepatocellular carcinoma in mice

**DOI:** 10.1371/journal.pone.0217009

**Published:** 2019-05-29

**Authors:** Shafinaz Abd Gani, Suleiman Alhaji Muhammad, Aminu Umar Kura, Farahnaz Barahuie, Mohd Zobir Hussein, Sharida Fakurazi

**Affiliations:** 1 Laboratory of Vaccine & Immunotherapeutics, Institute of Bioscience, Universiti Putra Malaysia, Serdang, Selangor, Malaysia; 2 Department of Biochemistry, Faculty of Biotechnology & Biomolecular Sciences, Universiti Putra Malaysia, Serdang, Selangor, Malaysia; 3 Department of Pharmacology, Faculty of Medicine, Bauchi State University, Gadau, Nigeria; 4 Faculty of Industry & Mining, University of Sistan and Baluchestan, Zahedan, Iran; 5 Materials Synthesis and Characterization Laboratory, Institute of Advanced Technology (ITMA), Universiti Putra Malaysia, Serdang, Selangor, Malaysia; 6 Department of Human Anatomy, Faculty of Medicine and Health Sciences, Universiti Putra Malaysia, Serdang, Selangor, Malaysia; Alexandria University, EGYPT

## Abstract

Researchers investigating cancer chemotherapy and management continue to search for agents that selectively kill malignant cells and leave healthy neighboring cells intact. Natural products provide relevant resources for anti-cancer drug discovery. However, the physicochemical properties of these compounds limit their efficient uptake and bioavailability. We introduced a nanocarrier system, namely, zinc-aluminum-layered double hydroxide (ZnAl-LDH) intercalated with protocatechuic acid. In this study, the efficacy and toxicity of protocatechuic acid intercalated in zinc aluminum-layered double hydroxide nanoparticles (PCA-ZnAl) against diethylnitrosamine/phenobarbital (DEN/PB)-induced hepatocellular carcinoma (HCC) in BALB/c mice was evaluated. HCC in male mice was induced by a single-dose intraperitoneal administration of DEN and was promoted by the introduction of PB via drinking water for 12 weeks. HCC induction was confirmed after the DEN/PB introduction period by measurement of the elevated level of serum α-feto protein (AFP). The results showed that the level of α-fetoprotein was significantly reduced in PCA-ZnAl (350±43.90 ng/mL), doxorubicin (DOX) (290±20.52 ng/mL) and ZnAl-LDH (390±19.65 ng/mL) treated animals compared to HCC mice treated with normal saline (580.4± 52.04 ng/mL). Superoxide dismutase (SOD), catalase (CAT), and glutathione (GSH) levels were significantly increased, whereas the level of lipid peroxidation was significantly decreased in HCC mice treated with DOX, PCA-ZnAl and ZnAl-LDH compared with those in HCC mice treated with saline. Restoration of hepatocyte morphology was observed following treatment that was comparable to that in the normal control group. Deterioration of hepatic cells and a significant increase of aspartate transaminase (AST), alanine transaminase (ALT), and alkaline phosphatase (ALP) were observed in the cancer-induced untreated group compared with that in the groups treated with nanoparticles. The histopathological features of the liver obtained from PCA-ZnAl-treated mice showed a uniform size with a similar distribution of the nuclear-cytoplasmic ratio and nucleus centrally located in the cytoplasm, similar to the normal liver cells. The results underscored the potential of PCA-ZnAl for the treatment of hepatocellular carcinoma.

## Introduction

Primary liver cancer is the fifth most common cancer in the world in men and the sixth most common in women worldwide [1]. Primary liver cancer is third most common cancer in developing countries among men after lung and stomach cancer. Approximately 60% of new cases of primary liver cancers are found in China, sub-Saharan Africa, Japan, and South-East Asia [2]. The burden of liver cancer in the Asia-Pacific region is substantial due to a high number of individuals suffering from chronic hepatitis B virus infection [3]. Chronic infection with hepatitis B and C viruses are the leading risk factors for liver cancer development [4]. The complexity of liver cancer susceptibility is largely attributed to the interaction between genes and the environment, and the interplay of environmental factors, including diet and lifestyle parameters [5]. The pathogenesis of liver cancer falls within the spectrum of inflammatory changes, cirrhosis, and cancerous cell formation [6].

Liver cancer is among cancers with a poor prognosis because cancer resists most chemotherapeutic agents [7]. In the early stage of tumor development, surgery is usually the recommended treatment if the malignant growth is limited to one area of the liver and the liver is otherwise healthy [8]. However, this is not always the case in most patients, due to late presentation and secondary metastasis, as well as because the entire liver is affected during the first diagnosis. The aim of liver cancer chemotherapy in most cases is to slow the disease progression and control the associated symptoms. Chemotherapy with the current available medications has limited benefits for hepatocellular cancer (HCC) and may cause more severe side effects, making patients suffer rather than cure their ailment [9].

The natural product protocatechuic acid (PCA) has been reported to control the progression of liver cancer, and it is a widely distributed and naturally occurring phenolic acid. Some preliminary studies have indicated the positive effect of PCA on lung, breast, liver, cervix and prostate cancer cell lines as determined by (4,5-dimethylthiazol-2-yl)-2,5-diphenyltetrazolium bromide and lactate dehydrogenase assays [10]. It has been previously reported that PCA possesses antioxidant and anti-inflammatory properties against hepatotoxicity in the rat by modulating stress signal transduction [11]. Similarly, PCA has also been shown to inhibit cancer cell metastasis by down-regulation of Ras/Akt/NF-kB pathway [12]. Since oxidative stress has been recognized as a key factor in the progression of hepatocarcinogenesis, the use of PCA may be an effective medication for liver cancer.

Previously, we had synthesized zinc aluminum-layered double hydroxide nanoparticles intercalated with PCA for cancer treatment. PCA release from the nanoparticles occurred in a sustained and controlled manner, and the thermal stability of the intercalated PCA was significantly enhanced compared with that of free PCA. The PCA-loaded nanoparticles inhibited the growth of human cervical, liver, and colorectal cancer cell lines significantly and exhibited no toxic effects toward normal fibroblast 3T3 cells after 72 h of treatment [13]. The use of layered double hydroxides (LDH) nanoparticles as a drug delivery system have received much intention in the field of biomedical sciences. These nanoparticles possess high positively charged surface which permits their easy diffusion through the cell membrane without any need for further positively charged groups compared to nanoparticles such as mesoporous silicates [14] Furthermore, the ability of these particles to remain in blood circulation could be certain and increase their potential to allow them to cross the blood-brain barriers due to their small particle size [14].

HCC induction in mice by chemicals remains the gold-standard preclinical model to test *in vivo* pharmacologic activity [15]. One of many advantageous of this model is that it mimics the injury-fibrosis-malignancy cycle, which is similar to HCC development in humans. In this model, HCC can be established in a 2-step process through the introduction of 2 types of carcinogenic compounds. First, induction was carried out with diethylnitrosamine (DEN), a genotoxic compound that induces DNA changes in the liver. Next, phenobarbital (PB), a chemical that enhances tumor formation after initiation by DEN was injected intraperitoneally (I.P.). In neonate BALB/c, introducing phenobarbital after DEN promotes the progression of hepatic adenomas via the induction of cytochrome P450 (by 100-fold). This cytochrome P450 significantly enhances the bioactivation of DEN in the liver, resulting in DNA-adduct formation through an alkylation mechanism. It has been suggested that, upon metabolic activation, DEN produces the promutagenic products O6-ethyl deoxy-guanosine and O4 and O6-ethyl deoxy-thymidine in the liver that are responsible for its carcinogenic effects [16]. Therefore, both steps facilitate the clonal expansion of preneoplastic cells.

In this study, the effect of PCA-ZnAl nanoparticles on DEN/PB-induced HCC in BALB/c mice was investigated. During the HCC induction periods, the serum alpha fetoprotein level was used as a biomarker to confirm the presence of HCC before the intervention.

## Materials and methods

### Chemicals

Diethylnitrosamine (DEN) was obtained from Sigma Aldrich (St. Louis, MO, USA) and phenobarbital (PB) capsules were obtained from The Government Pharmaceutical Organization (Bangkok, Thailand). The alpha fetoprotein ELISA kit and doxorubicin hydrochloride were purchased from Cusabio Technology (Taiwan) and Toronto Research Chemicals Inc. (Toronto, Canada). Doxorubicin hydrochloride was purchased from Toronto Research Chemicals Inc. (Toronto, Canada). PCA-ZnAl-IEX was freshly prepared using ion exchange method in Materials Synthesis and Characterization Laboratory, Institute of Advanced Technology, Universiti Putra Malaysia. A thorough characterization of the nanocomposites was carried out before the in vivo study.

### Animal preparation

Fifteen-day-old male BALB/c mice were obtained from the Animal Resources Unit, Faculty of Veterinary Medicine, Universiti Putra Malaysia. The mice were housed in five animals/cage with woodchip bedding. The mice were marked by the tail for identification and were maintained under standard conditions of temperature (25 ± 2°C), relative humidity (70 ± 5%) and a 12-hour light-dark cycle. The animals were fed with standard mouse pellets and tap water *ad libitum* throughout the experiments. The research protocol was approved by the Universiti Putra Malaysia Institutional Animal Care and Use Committee (UPM/IACUC/AUP-R35/2014).

### Induction of hepatocellular carcinoma in BALB/c mice

Forty male neonatal BALB/c mice were injected with 10 μL of 50 μg/g body weight of DEN intraperitoneally. Female mice were excluded from the study. Following DEN injection, the mice were returned to the mothers to continue feeding. Upon weaning (at 28 days old), the mice were housed into five groups (5 animal/cages), and 500 mg/L of phenobarbital (PB) was introduced into their drinking water for 12 weeks. The drinking water containing PB was changed daily, and intake was allowed at *ad libitum*. The normal control group containing 10 BALB/c mice was allowed to have normal drinking water. Power calculation was performed a priori using GPower statistical software with a power of approximately 80% and an alpha of 0.05. Four mice died from cardiac injury during the induction period. The body weights were monitored weekly throughout the induction period. At the end of 12 week PB treatment, animals were randomized into five groups; non-induced (10 animals) and HCC group (4 groups of 9 animals each). DOX-treated mice serve as positive control.

### Confirmation of the presence of hepatocellular carcinoma

At week 12 after the initiation and induction processes, three mice each were randomly selected from the normal and induced groups and blood was withdrawn via cardiac puncture. The serum alpha-fetoprotein (αFP) level, which is a tumor marker for HCC, was measured using an ELISA kit (Cusabio Technology, Taiwan). The level of αFP was set at 150 ng/mL for the induction of liver tumor. This level has been previously reported to induce primary liver cancer and teratocarcinoma [17].

### Animal treatment with nanoparticles

Following confirmation of the level of αFP (≥ 150 ng/ml), the mice were divided randomly into five groups. Consequently, freshly prepared nanoparticles were administered intraperitoneally, every other day for 28 days because the 1/2 life of the nanocomposite was 3000 min (2.08 days). Observation of physical changes particularly in the testicular area and behavior of mice during the HCC induction & after the nanoparticles treatment were recorded.

Group 1 comprised normal BALB/c mice (non-induced and treated with 0.02 mL of saline/mouse). Group 2 was DEN/PB-induced HCC treated with saline (0.02 mL/mice) only. Group 3 was DEN/PB-induced HCC administered doxorubicin (2 mg/kg body weight), Group 4 was DEN/PB-induced HCC treated with 100 mg/kg body weight of PCA-ZnAl, and Group 5 was DEN/PB-induced HCC administered 100 mg/kg body weight of Zn/Al nanoparticles in a volume of 0.02 mL of saline/mouse via intraperitoneal injection. Doxorubicin and DEN were adjusted to pH ~7 to prevent tissue necrosis and painful sensation at the site of injection. The concentration of 2 mg/kg of DOX was adopted from the previous study [18], whereas that of the nanocomposite was selected based on the result of IC_50_ from the *in vitro* study.

### Sacrifice of mice and collection of organs

Twenty-four hours after the last treatment, the mice were sacrificed with xylazine and ketamine at a dose ratio of 80 and 10 mg/kg, respectively, via IP injection, and blood samples were collected via cardiac puncture. Major organs, including the heart, kidney, liver, spleen, lung, and testis were collected, rinsed with cold saline, blotted dry, and weighed.

### Histopathology

The heart, kidney, liver, spleen, lung, and testis were excised, weighed, and checked for any macroscopic abnormality due to the treatment regimen. Tissues from these organs were fixed in 4% paraformaldehyde for histological analysis. The tissues were later subjected to standard tissue processing and were embedded in paraffin blocks. The tissues were microsectioned into 5-μm thick pieces and were mounted onto glass slides. Hematoxylin and eosin (H&E) staining was performed on the slides, and they were viewed using an optical microscope (Olympus FSX-100; Tokyo, Japan).

### Serum biochemical analysis

Blood collected via cardiac puncture was placed in a plain 2-mL centrifuge tube and was incubated for 30 min at room temperature before centrifuging at 1000 × g for 10 min (Eppendorf 5810R; Hamburg, Germany). Serum enzyme activities, including those of aspartate transaminase (AST) and alanine transaminase (ALT), as well as the urea, bilirubin, creatinine, chloride, sodium, and potassium levels, were measured using an automated biochemistry analyzer (Hitachi 902; Tokyo, Japan).

### Determination of the antioxidant status of the liver homogenates

The frozen liver tissue samples were thawed prior to the addition of ice-cold saline. The mixtures were then homogenized, and the protein concentrations were determined using the Pierce 660-nm Protein Assay Kit (Thermo Fisher Scientific, Rockford, IL, USA) according to the manufacturer’s instructions. Spectrophotometric assay kits were used to measure the superoxide dismutase (SOD) (Cayman-706002), catalase (CAT) (Cayman-707002) and glutathione (GSH) (Cayman-703002) activities, as well as the thiobarbituric acid reactive substances (TBARS) (Cayman-10009055) level, in the prepared homogenates.

### Determination of the nanoparticle distribution in relevant tissues and serum

The distribution and deposition of PCA-ZnAl were determined in the organs and serum isolated from the treated mice. Tissue digestion was carried out as previously described [19]. Briefly, approximately 0.1 g of tissue or 500 μL of serum acquired from each animal was added to a mixture of ultrapure-grade nitric acid and H_2_O_2_ at a ratio of 6:1 for digestion using a microwave digester (Anton Paar, Austria) [20]. Before the procedure was conducted, all glassware used were rinsed and soaked in 10% (v/v) HNO_3_ overnight. They were then rinsed with deionized water and dried before use. After the digestion process, the cap of each high-pressure container was loosened to allow the remaining nitric acid to evaporate until the solution turned from yellowish to colorless. Next, ultrapure water was added to the colorless solution until the volume reached 10 mL before the solution was subjected to zinc concentration analysis using atomic absorption spectroscopy (AAS; Thermo Elemental X7; Thermo Electron Co., Waltham, USA).

### Statistical analysis

Statistical analysis of the data was performed using SPSS software version 20.0. The mean values and standard deviations (SDs) of both the treated and control groups were calculated (n = 8–10). One-way ANOVA was used to compare the groups; where significant differences existed, Tukey post-hoc test was used to compare the differences. *p*<0.05 was set as the statistically significant level.

## Results

### Characterization of nanocomposites

Intercalation of PCA resulted in moderate trapping efficiency (30.84 ± 0.36%). The transmission electron microscopy analysis indicated nanocomposites had a spherical plate-like shape with size ranging from 20 to 60 nm. Dynamic light scattering assay revealed that the size of the nanocomposites formed was 92.29 ± 4.21nm particle size with +2.43±0.50 mV which was an optimum value for cellular uptake. The Nanodrug demonstrated excellent drug retention properties (79% in 6706 min, 1/2 life 3000 min) compared to the respective rapid burst release of the pure drug (100% in 12 min). PCA-ZnAl-IEX showed superior cytotoxic activities toward HepG2 compared to the other cell line tested, having the lowest IC_50_ of 20.5 μg/ml in the in vitro setting.

### Confirmation of the presence of hepatocellular carcinoma in mice induced with DEN/PB

**Fig 1:**
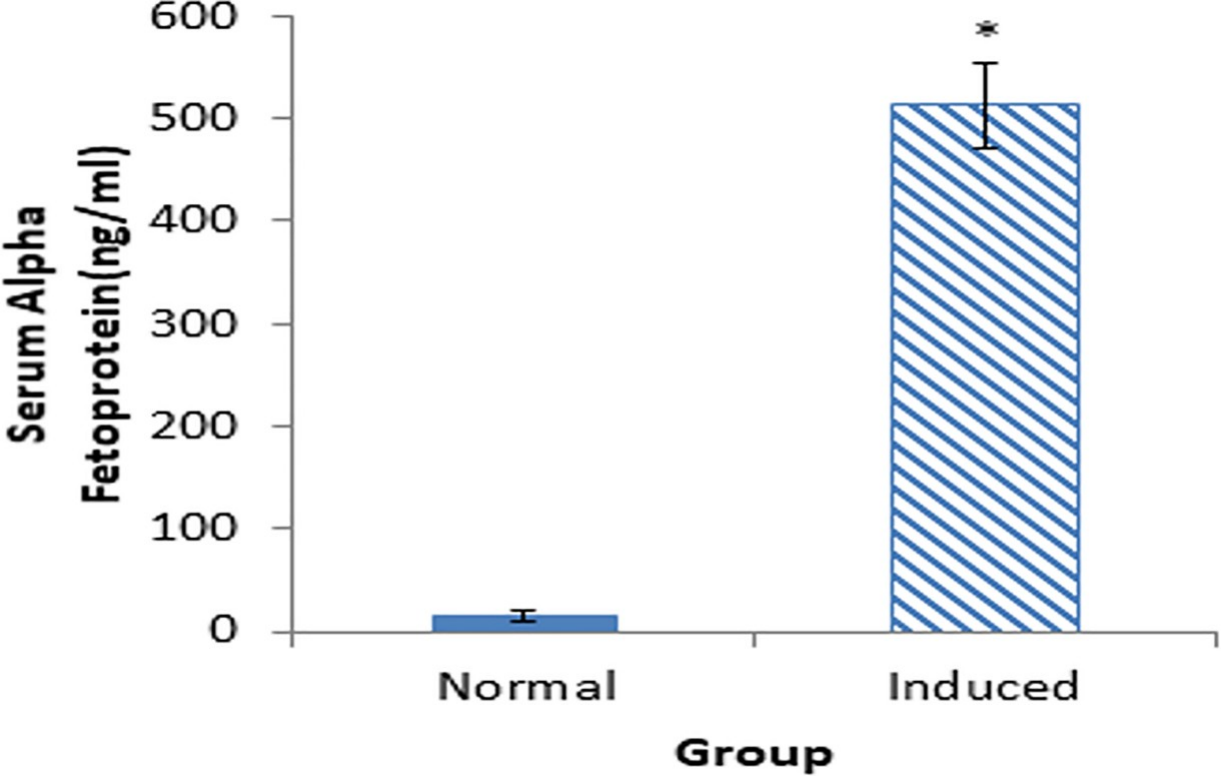
Level of alpha fetoprotein in the serum of normal and DEN/PB-induced HCC mice. The data are expressed as mean ± SD. *p < 0.05 is significantly different when compared with normal mice using unpaired t test.

The level of α-FP was analyzed to confirm the presence of HCC in the induced mice after 12 weeks of induction. Prior to the induction of cancer using DEN/PB, blood was collected and subjected to the α-FP assay. The level of α-FP was 15.23±10.83 ng/mL in the serum of the mice before induction. However, following induction with DEN/PB, the level of α-FP (512±40.83 ng/mL) was significantly increased in mice that were administered with both DEN and PB (Fig 1).

### Changes in the body weight after repeated drug treatment

The body weights of the animals subjected to different treatments are shown in Table 1. Mice in the normal group showed a sustained body weight throughout the treatment period. Induced mice treated with saline showed a 10.51% body weight loss during the treatment period, although the decrease was not significantly different compared with that among the weeks. The group of mice administered DOX and ZnAl-LDH also maintained their body weight during the experimental period. However, the group administered PCA-ZnAl showed a significant body weight increase at the end of 4 weeks of treatment.

**Table 1:** Summary of the weight changes in mice during the drug treatment period.

Group		Weight (g)		
	Week 1	Week 2	Week 3	Week 4
Normal	24.69±1.28^a^	24.75±1.34^a^	25.13±1.15^a^	25.38±1.40^a^
Induced/Saline	18.94±1.69^a^	17.72±1.63^a^	17.70±1.48^a^	16.95±1.60^a^
Induced/DOX	20.45±1.28^a^	20.40±0.38^a^	20.65±0.19^a^	20.95±0.13^a^
Induced/PCA-ZnAl	21.05±1.49^a^	22.80±1.00^b^	23.55±0.84^b^	25.10±0.86^c^
Induced/ZnAl-LDH	21.10±1.56^a^	20.35±1.03^a^	20.65±0.71^a^	21.55±1.58^a^

The data are expressed as means ± SD, n = 8/10. Values across the table with the same superscript are not significantly different using one-way ANOVA followed by Tukey’s post-hoc test.

### Testicular features of the BALB/c mouse model of hepatocellular carcinoma

**Fig 2:**
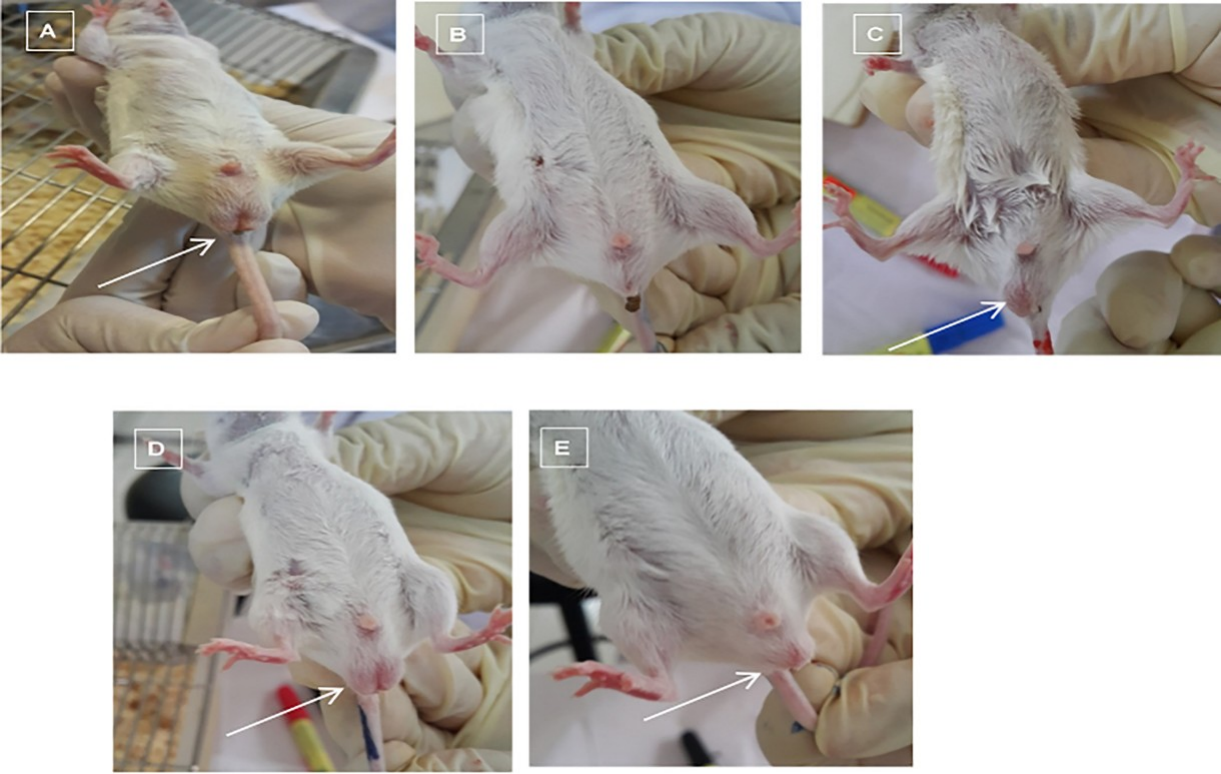
Macroscopic appearance of the testicular area of male BALB/c mice after 4 weeks of drug treatment. A) Normal group, B) induced/saline-treated group, C) induced/DOX-treated group, D) induced/PCA-ZnAl-treated group, and E) induced/ZnAL-LDH-treated group. In the normal group, the testicular area was intact. However, in the induced/saline, induced/DOX, and induced/ZnAl-LDH groups, the testicular area looked abnormal with shrinkage of the scrotum and distortion of the scrotum; in the induced/PCA-ZnAl group, the scrotum appeared largely similar to that of the normal control group.

A decrease in the size of both the scrotum and testicles was observed in HCC mice treated with saline (Fig 2B). The decline in the size of the scrotum resulted from undescended testicles suspected to be crytorchidism syndrome. Following post mortem, the testicles were located in the groin adjacent to the bladder. However, in cancer-induced mice treated with DOX, a similar observation was made, where the decline in the size of the scrotum and testicles was observed, but with less severity (Fig 2C). In HCC animals treated with ZnAl-LDH, the genital area was observed to be similar to that of control, but with a smaller size (Fig 2E). Interestingly, the testes in the PCA-ZnAl-treated mice (Fig 2D) were observed to be mobile in the sac in the genital region inside the scrotum with similar features to those of the control (Fig 2A).

### Macroscopic changes in the liver of the BALB/c mouse model of hepatocellular carcinoma

**Fig 3:**
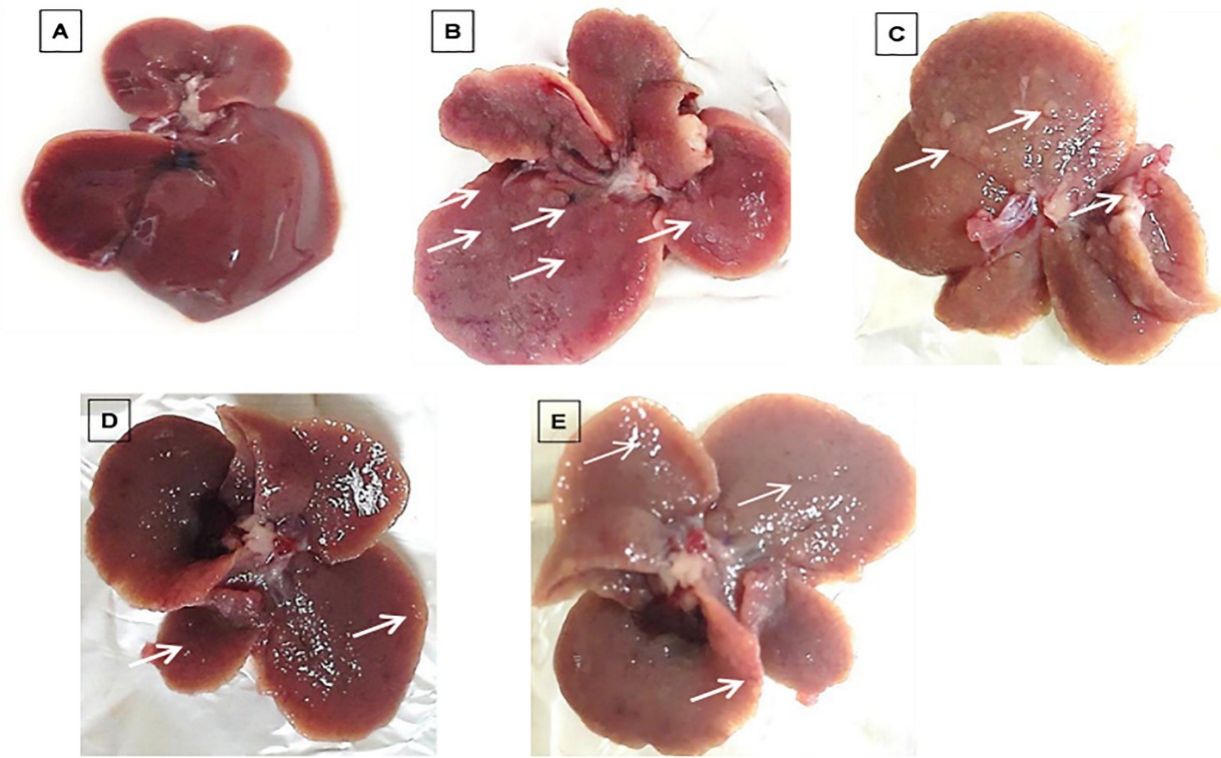
Liver features of mice 28 days post drug treatment. Macroscopic features of the liver from A) normal mice, B) induced/saline-treated group, C) induced/DOX-treated group, D) induced/PCA-ZnAl-treated group, and E) induced/ZnAl-LDH-treated group. A smooth and regular hepatic surface can be observed in the normal liver. Several small nodules are visible in all mice induced with DEN/PB.

The appearance of the liver in the control group was normal, and no nodules were detected macroscopically (Fig 3A). Macroscopic hepatic changes were noted in all DEN/PB-induced groups (Fig 3B–3E). After 28 days of treatment, all DEN/PB-induced and saline-treated mice revealed hepatomegaly and small nodules with a rough surface on the liver. Small nodules were also observed on the liver surface of mice treated with DOX, PCA-ZnAl, and ZnAl-LDH after 28 days of treatment.

### Weights of the heart, kidney, liver, lungs, spleen, and testis of BALB/c mice after repeated drug treatment

**Fig 4:**
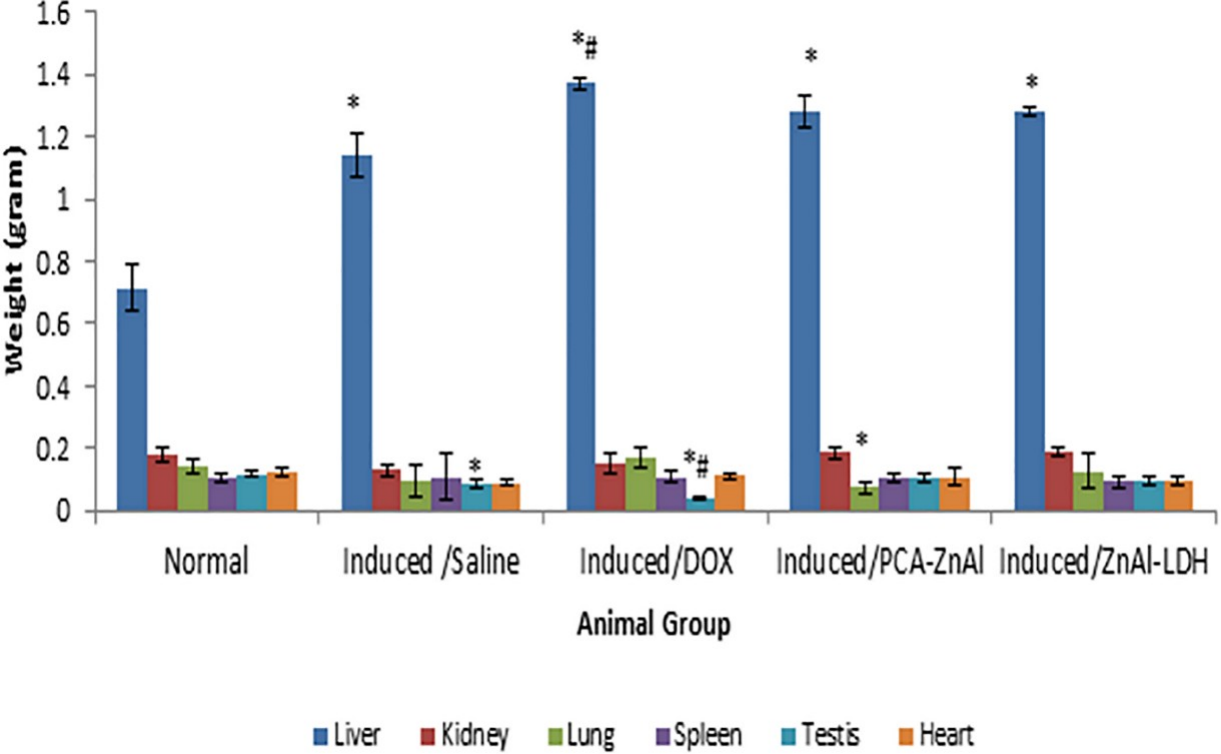
Weight of organs in normal and DEN/PB-induced HCC mice 28 days post drug treatment. The values are expressed as the mean ± SD, n = 10. *p< 0.05 indicates a statistically significant difference compared with the normal group. #*p*< 0.05 indicates a statistically significant difference compared with the induced/saline group.

Fig 4 shows the weights of organs isolated from mice treated with nanoparticles after 28 days. There was no significant change in the weights of the kidney, heart and spleen between DEN/PB-induced hepatocellular carcinoma mice and normal control mice. The weight of the liver was significantly increased in HCC mice treated with DOX, PCA-ZnAl, and ZnAl-LDH compared with that in the normal group. Similarly, a significant increase in the weight of the liver was observed in DOX-treated mice compared with that in mice induced and treated with saline. Furthermore, there was a significant decrease in the weight of the testis of induced mice treated with saline and DOX compared with that of normal control mice. There was also a significant decrease in the weight of the lung of HCC mice treated with PCA-ZnAl compared with that of normal control mice.

### Level of alpha-fetoprotein (α-FP) in HCC mice treated with nanoparticles

**Fig 5:**
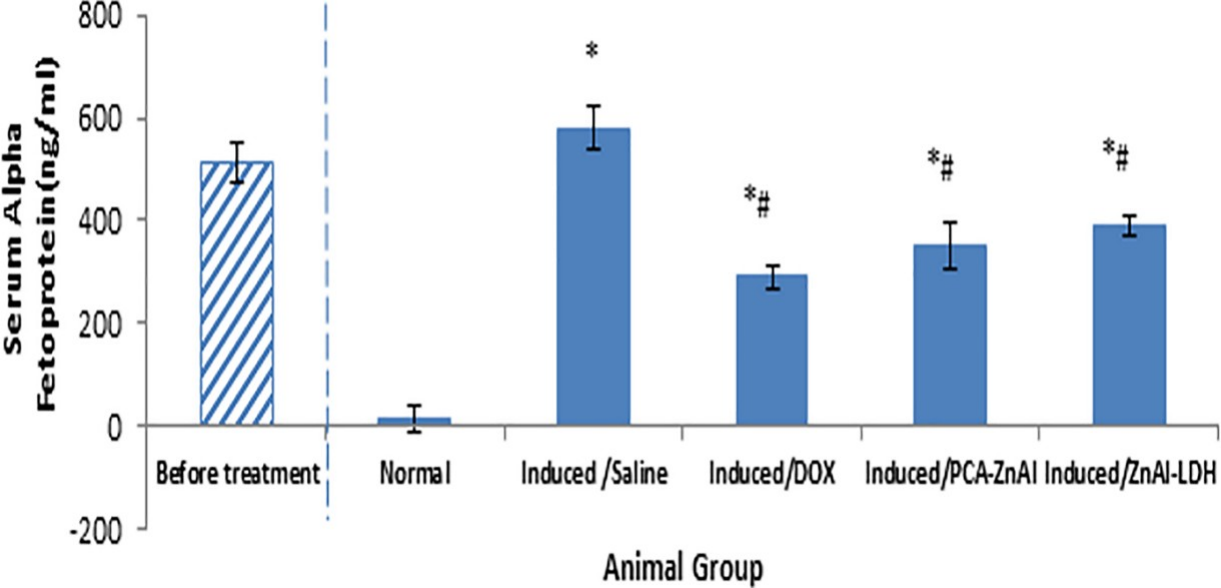
Level of alpha-fetoprotein in the serum of DEN/PB-induced HCC mice after treatment with nanoparticles. All the data are expressed as means ± SD, n = 8. *p<0.05 indicates a statistically significant difference compared with the normal group. #*p*< 0.05 indicates a statistically significant difference compared with the induced/saline group using one-way ANOVA.

The level of α-FP, a tumor marker (Fig 5), was barely detectable in normal mice throughout the study period. Following induction with DEN/PB, the level of α-FP was elevated to 512±40.83 ng/mL. When the presence of HCC was confirmed, the mice were randomized into treatment groups. After 28 days of treatment, the level of α-FP in the DOX, PCA-ZnAl, and ZnAl-LDH groups was significantly decreased compared with that in the saline-treated control (580.4±52.04 ng/mL). The level of α-FP was decreased to 290±20.52 ng/mL, 350±43.90 ng/mL and 390±19.65 ng/mL in mice treated with DOX, PCA-ZnAl and ZnAl-LDH, respectively.

### Level of serum liver enzymes in HCC mice

As shown in Table 2, induction with DEN/PB for 12 weeks and subsequent treatment with saline significantly elevated the activities of ALT and AST compared with those in normal mice, suggesting possible impediment of the functional activity of the liver following DEN/PB administration. However, treatment with PCA-ZnAl and ZnAl-LDH showed a significant reduction in the level of serum ALT compared with that in mice treated with saline. Similarly, the level of AST was also found to be reduced in the group treated with PCA-ZnAl and ZnAl-LDH, although the reduction was not significantly different when compared with mice treated with saline. The activity of ALP was significantly reduced in the DOX-treated group compared with that in the saline-treated control group.

**Table 2:** Serum liver markers in HCC mice after 28 days of drug treatment.

Serum Marker Levels								
Group	ALT (U/L)	AST (U/L)	ALP (U/L)	Total Protein (g/L)	Total Bilirubin (μmol/L)	Albumin (g/L)	Globulin (g/L)	Albumin/ Globulin Ratio
**Normal**	33.90±2.40	116.23±12.03	94.67±13.90	93.27±1.34	0.40±0.61	37.77±0.42	15.50±1.76	2.45±0.31
**Induced/Saline**	142.83±27.08*	221.87±51.68*	193.00±7.78*	67.00±6.26*	1.97±0.35*	33.23±1.56*	33.77±2.97*	1.02±0.26*
**Induced/DOX**	91.30±44.50*	225.23±52.44*	147.33±14.84*^#^	61.23±8.89	1.50±0.14*	32.50±2.89*	28.73±3.04*	1.17±0.29*
**Induced/PCA-ZnAl**	77.10±23.47^#^	154.20±28.22	173.67±17.62*	65.23±2.06*	0.70±0.26^#^	35.03±0.61	20.20±1.56*^#^	1.74±0.12*^#^
**Induced/ZnAl-LDH**	81.30±118.50^#^	158.37±22.35	180.33±15.56	67.53±1.91*	0.83±0.28^#^	34.03±1.92	23.50±0.52^#^	1.44±0.09*^#^

All the data are expressed as the means ± SD, n = 8.

*p< 0.05 indicates a statistically significant difference compared with the normal group.

#*p*< 0.05 indicates a statistically significant difference compared with the induced/saline group using one-way ANOVA followed by Tukey’s post-hoc test. ALT-alanine aminotransferase, AST-aspartate aminotransferase, ALP-alkaline phosphatase.

There was a significant reduction in the level of total bilirubin, globulin and albumin/globulin ratio in HCC mice treated with PCA-ZnAl or ZnAl-LDH compared with that in untreated HCC controls. The levels of total protein and albumin were not significantly different compared with those in the untreated HCC controls. Furthermore, a significant reduction in the levels of total protein, albumin, and albumin/globulin ratio were observed in the HCC mice compared with those in the normal control mice. The total bilirubin level in the HCC mouse treated groups and untreated control group was significantly increased compared with that in the normal control group with the exception of PCA-ZnAl, where the difference was not statistically significant.

### Level of serum electrolytes, creatinine, and urea in HCC mice treated with nanoparticles

**Fig 6:**
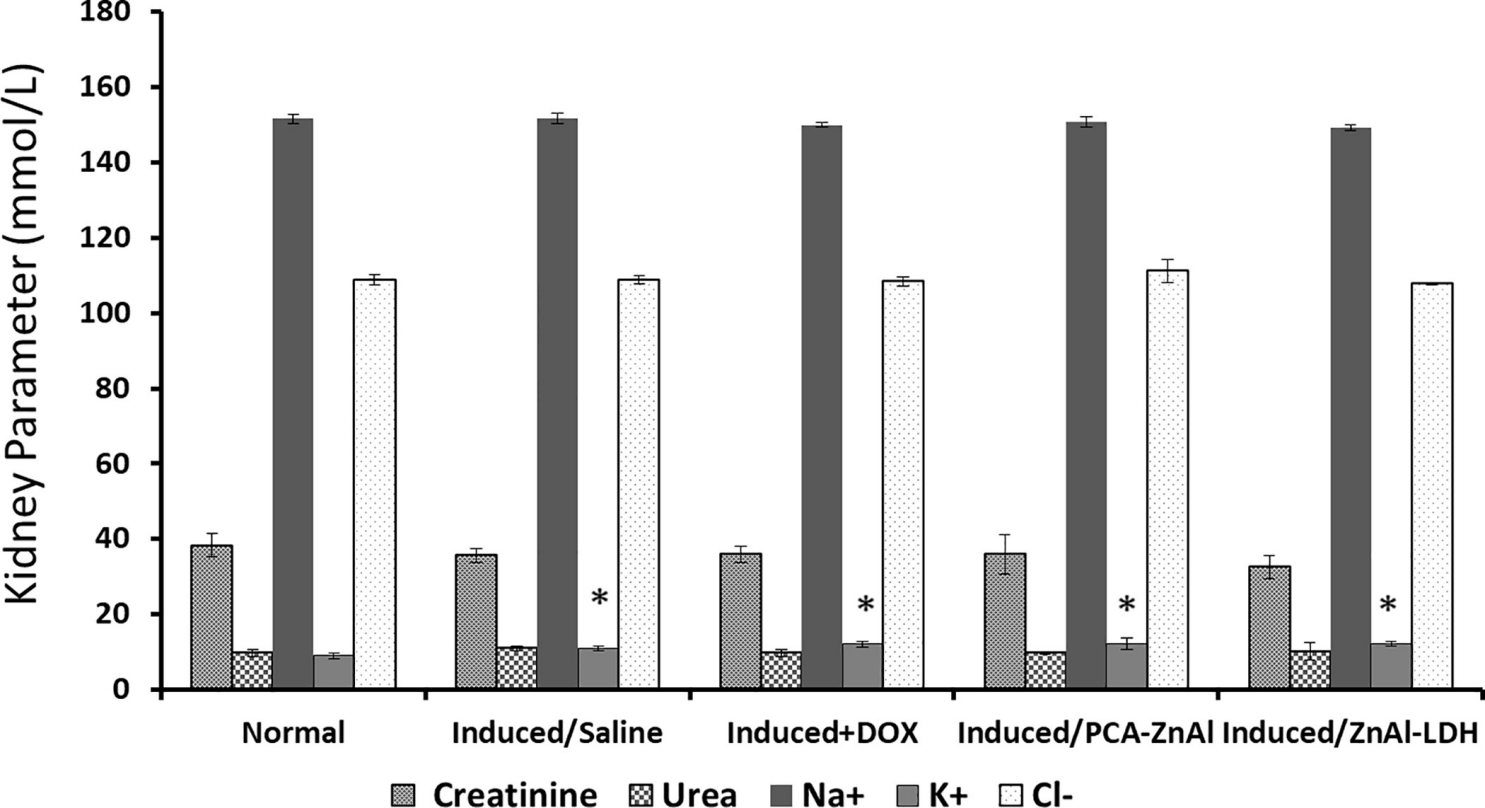
Effect of drug treatment on serum electrolytes, creatinine, and urea in mice after 28 days of treatment. All of the data are expressed as means ± SD, n = 8. *p< 0.05 indicates a statistically significant difference compared with the normal control.

The analysis of serum electrolytes, creatinine, and urea levels in HCC mice was carried out after 28 days of treatment with saline, DOX, PCA-ZnAl, and ZnAl-LDH. The results showed that treatment of HCC mice with DOX, PCA-ZnAl, and ZnAl-LDH caused no significant change in the level of creatinine, urea, Na^+^ and Cl^-^ compared with that in the normal control mice (Fig 6). However, there was a significant increase in the level of K^+^ in HCC mice treated with DOX, PCA-ZnAl, and ZnAl-LDH compared with that in the normal control mice. Similarly, the level of K^+^ was significantly higher in HCC mice treated with normal saline than in the normal control mice.

### Antioxidant status evaluation

It has been reported that oxidative stress plays key roles in the induction and development of hepatocarcinoma. The antioxidant status of HCC mice treated with DOX, PCA-ZnAl, and ZnAl-LDH is depicted in Table 3. The TBARS level was measured in liver homogenates to evaluate hepatic lipid peroxidation in mice induced with HCC and treated with nanoparticles. The results showed that treatment of HCC mice with DOX, PCA-ZnAl, and ZnAl-LDH significantly decreased the level of TBARS in the liver homogenate compared with HCC mice treated with normal saline. Furthermore, there was no significant difference in the level of TBARS between the DOX, PCA-ZnAl, or ZnAl-LDH group and normal control group. The results suggest that treatment significantly attenuated the level of lipid peroxidation.

**Table 3:** Effects of various treatments on the hepatic antioxidant system in normal and DEN/PB-induced mice.

Group	TBARS (mole/g tissue)	SOD (μg/mL)	CAT (μM/g tissue)	GSH (mg/g tissue)
**Normal**	322.75±15.91	110.15±5.45	319.20±2.83	1.142±0.003
**Induced /Saline**	988.17±35.47*	73.74±2.17*	165.2±5.66*	0.089±0.002*
**Induced/DOX**	305.17±20.13^#^	100.25±3.26^#^	272.97±14.78*^#^	0.875±0.007*^#^
**Induced/PCA-ZnAl**	302.83±12.06^#^	105.53±3.78^#^	294.73±5.60*^#^	0.894±0.103*^#^
**Induced/ZnAl-LDH**	365.5±1.41*^#^	93.82±4.73*^#^	197.20±22.62*^#^	0.362±0.05*^#^

The values represent the means ± SD, n = 8.

*p < 0.05 indicates a statistically significant difference compared with the normal group.

#*p* < 0.05 indicates a statistically significant difference compared with the induced/saline group. SOD-superoxide dismutase, CAT-catalase, GSH-glutathione, TBARS- thiobarbituric acid reactive substances.

The results of SOD, GSH and CAT indicated that the administration of DEN/PB to mice significantly decreased the antioxidant status, but treatment with DOX, PCA-ZnAl, or ZnAl-LDH significantly improved the level of these antioxidant markers (Table 3). The levels of these antioxidant markers were markedly increased in the treated groups compared with those in HCC mice treated with saline. Furthermore, the levels of CAT, GSH and SOD were increased in PCA-ZnAl-treated group compared to the group treated with DOX or ZnAl-LDH. The SOD level in HCC mice treated with PCA-ZnAl and DOX was similar when compared with the normal control, however, a significant decrease was observed in SOD activity in the ZnAl-LDH group compared to normal control. Overall, treatment of HCC mice with DOX, PCA-ZnAl, and ZnAl-LDH significantly improved the antioxidant status and decreased the oxidative stress with PCA-ZnAl, displaying superior effect.

### Histology of the liver

**Fig 7:**
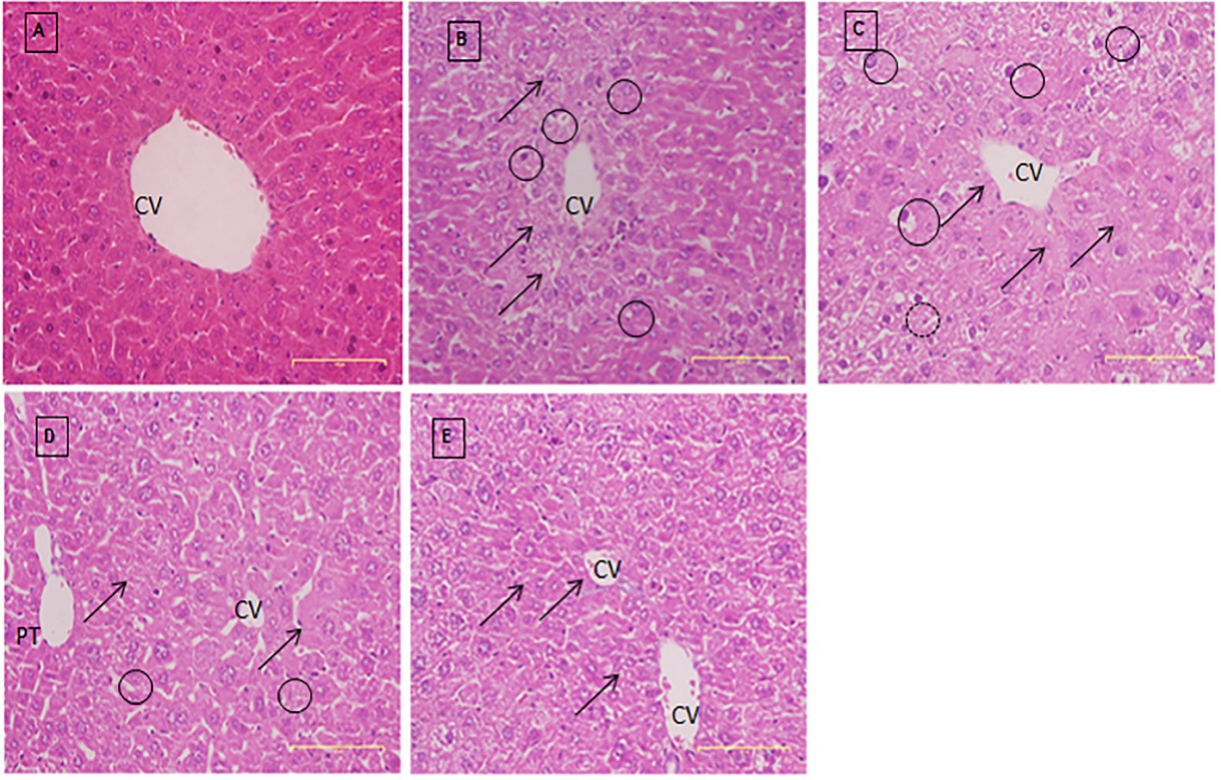
Photographic sections of H & E staining of normal and experimental mouse liver. Histology sections of the liver obtained from the A) normal group, B) induced/saline group, C) induced/DOX group, D) induced/PCA-ZnAl group and E) induced/ZnAl-LDH group. Abbreviations: Portal triad (PT), central vein (CV). The hepatic lobular array was well maintained with a central vein at the center, as shown in the normal group (A). These features were altered in all the DEN/PB-induced mice (B-E). Extensive cell swelling (circle) and single-cell necrosis (arrow) can be observed. Magnification100×.

A well-maintained hepatic lobular array with a well-preserved architecture of central vein (CV) at the center is shown in the control, and a normal array of sinusoids and radiating cords of hepatocyte were seen (Fig 7A–7E). The hepatocytes were polyhedral in shape, and their cytoplasm was granulated with small uniform nuclei (Fig 7A).

These features were shown to be altered in all HCC-induced mice. Extensive cell swelling and single-cell necrosis were observed in liver sections from cancer-induced mice that were treated with saline. Necrotic cells were observed with basophilic nuclei and dark cytoplasm. Dysplastic hepatocytes with enlarged nuclei (karyomegaly) and multiple nucleoli were also detected in the liver sections obtained from this group of mouse livers (Fig 7B).

Vacuolated hepatocytes and hyaline globules were noticed in cancer-induced mice that were treated with DOX. Cells without nuclei were spotted surrounding the CV area (Fig 7C). In liver sections of cancer-induced mice treated with PCA-ZnAl, the morphology of hepatocytes was relatively preserved. Hepatocytes of mice that were DEN/PB-induced and treated with PCA-ZnAl (Fig 7D) exhibited almost similar features to the liver of normal mice (Fig 7A). The numbers of necrotic cells and dysplastic hepatocytes were reduced, showing an advanced pattern of recovery (Fig 7D). Additionally, active Kupffer cells were observed. Furthermore, eosinophilic granular cytoplasm, prominent nucleoli, and rounded nuclei were observed in mice treated with Zn-LDH (Fig 7E).

### Histology of the testis

**Fig 8:**
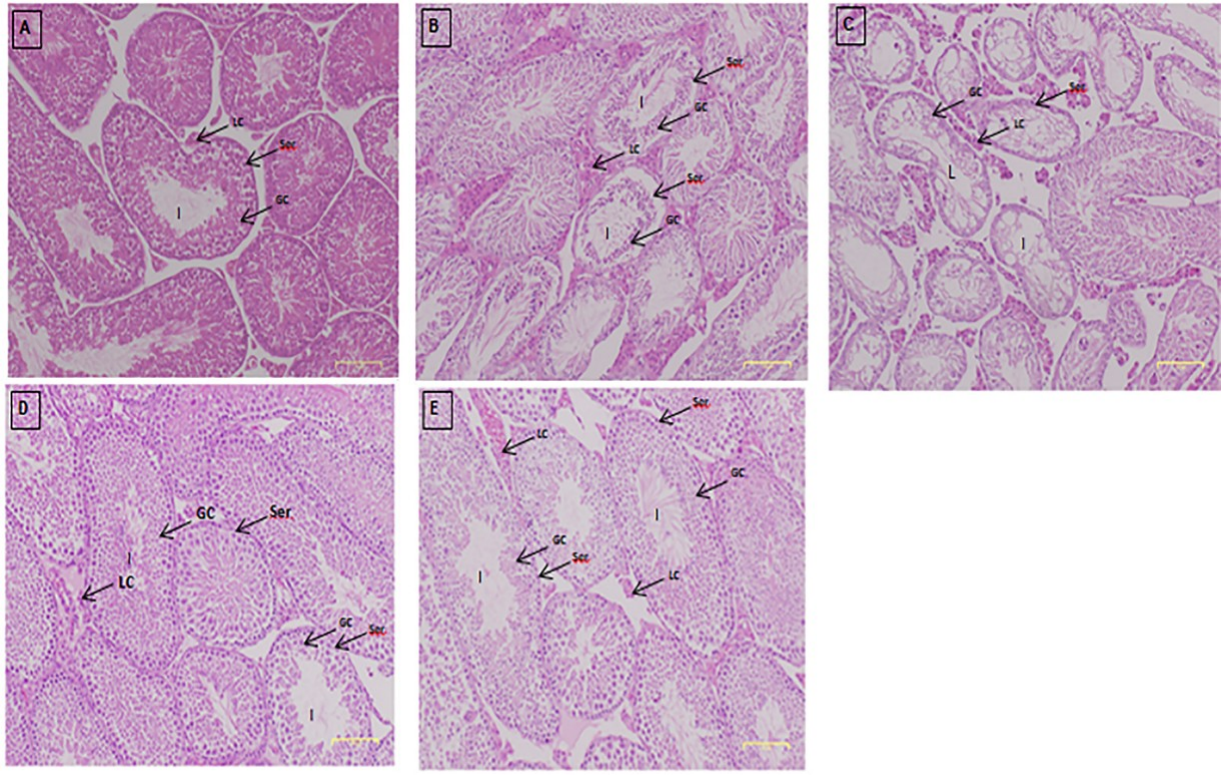
Histology of the testis tissue of mice after 28 days of drug treatment stained with H & E. Photomicrograph of testis sections in the A) normal group, B) induced/saline group, C) induced/DOX group, D) induced/PCA-ZnAl group and E) induced/ZnAl-LDH group. The Leydig (LC) interstitial cells and Sertoli cells (Ser) were clearly displayed in (A), and the germ cells within the Sertoli region are at different stages of development. Fewer differentiating germ cells (GC) were seen in (B); however, in C, D and E, the germ cells are very few, with more Leydig cells and interstitial fibrosis in C than in D or E. The lumen (L) of the seminiferous tubule appears empty in B and C compared with that of the normal group (A). Magnification, 40×.

Fig 8A shows an intact architecture of seminiferous tubules of testicular tissue where the germ cells within the Sertoli region is at different stages of development. Compared with control mice, the tissue from mice induced with DEN/PB and treated with the saline group showed complete disorganization of these tubules. These agents caused seminiferous tubule damage as shown by the histopathological changes, including disruption of the basement membrane and disorganized arrangement of germ cells in seminiferous tubules, leaving contracted tubules lined only by fewer Sertoli cells. The lumenal part of the tubule contained slough with almost no spermatozoa. The interstitial spaces between the seminiferous tubules appeared wider than the control (Fig 8B–8E).

### Histology of the lung

**Fig 9:**
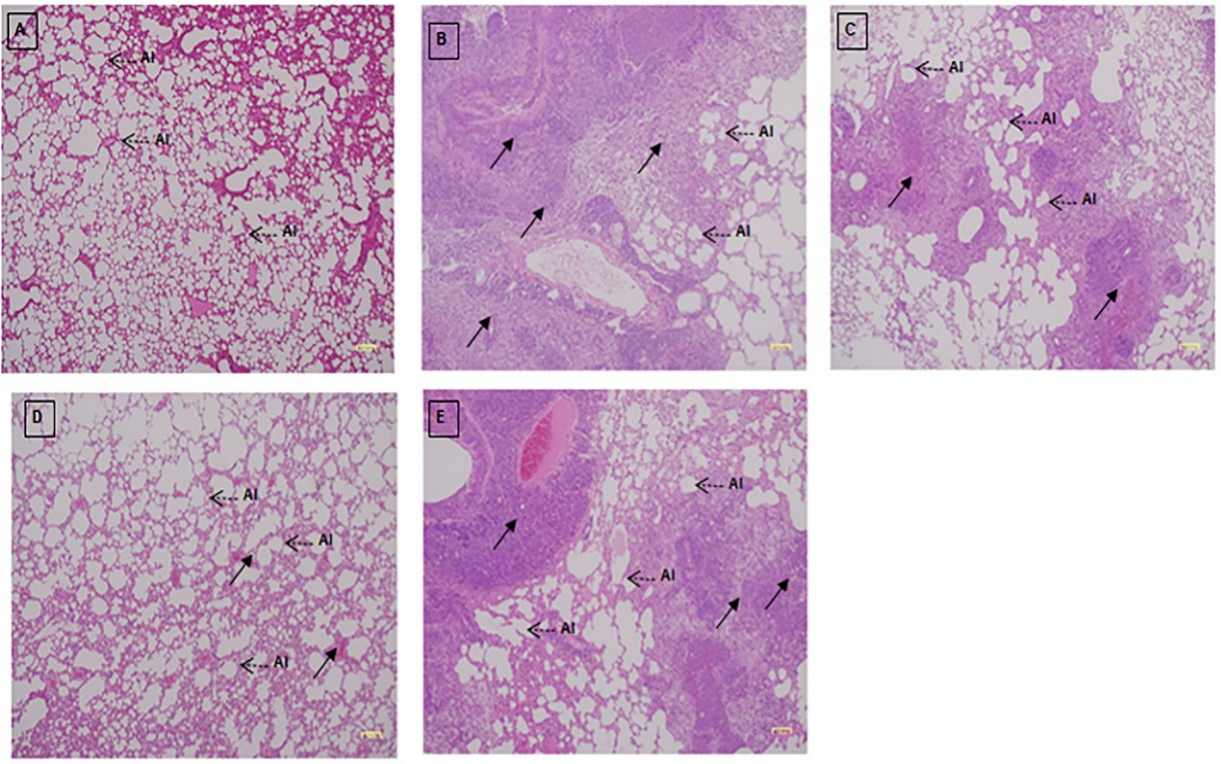
Histology of lung tissue after H & E staining in mice 4 weeks post treatment. Histology sections of the lungs were obtained from the A) normal group, B) induced/saline group, C) induced/DOX group, D) induced/PCA-ZnAl group and E) induced/ZnAl-LDH group. For (B), (C) and (E), the lung was covered with collapsed and disfigured alveoli (Al), severely ruptured air sacs and alveoli and a very few intact alveoli with cellular infiltration (arrow). Regarding (D), treatment with PCA-ZnAl causes the wall between the alveoli to thicken with less severe infiltration. Magnification, 40×.

Histopathological features of the lung tissue of the normal group and DEN/PB-induced mice treated with PCA-ZnAl displayed a fine lace appearance due to the presence of thin-walled alveoli in the normal mouse lung tissue (Fig 9A and 9D). There is thickening of the alveoli in DEN/PB-induced mice treated with saline, DOX, and ZnAl-LDH with hyperinfiltration of inflammatory cells and a generalize distortion of normal lung tissue architecture (Fig 9B, 9C and 9E).

The alveoli in Fig 9A that represent normal mice comprised a single layer of squamous epithelium, where the alveoli sacs are small and interconnected with abundant alveoli. However, the collapse of the alveolar sac into a larger ballooning sac with a thickened alveolar membrane containing more than one cell is detected in all DEN/PB-induced groups treated with saline, DOX, PCA-ZnAl, and ZnAl-LDH (Fig 9B–9E). In Fig 9B, 9C and 9E, the lung tissues were covered by collapsed and disfigured alveoli, while the ruptured epithelial alveoli wall contributed to the formation of dense cavity space filled with inflammatory cell aggregates. DEN/PB-induced and PCA-ZnAl-treated mice displayed mild lung tissue damage with few inflammatory infiltrates and some alveoli wall breakage (Fig 9D).

### Histology of the kidney

**Fig 10:**
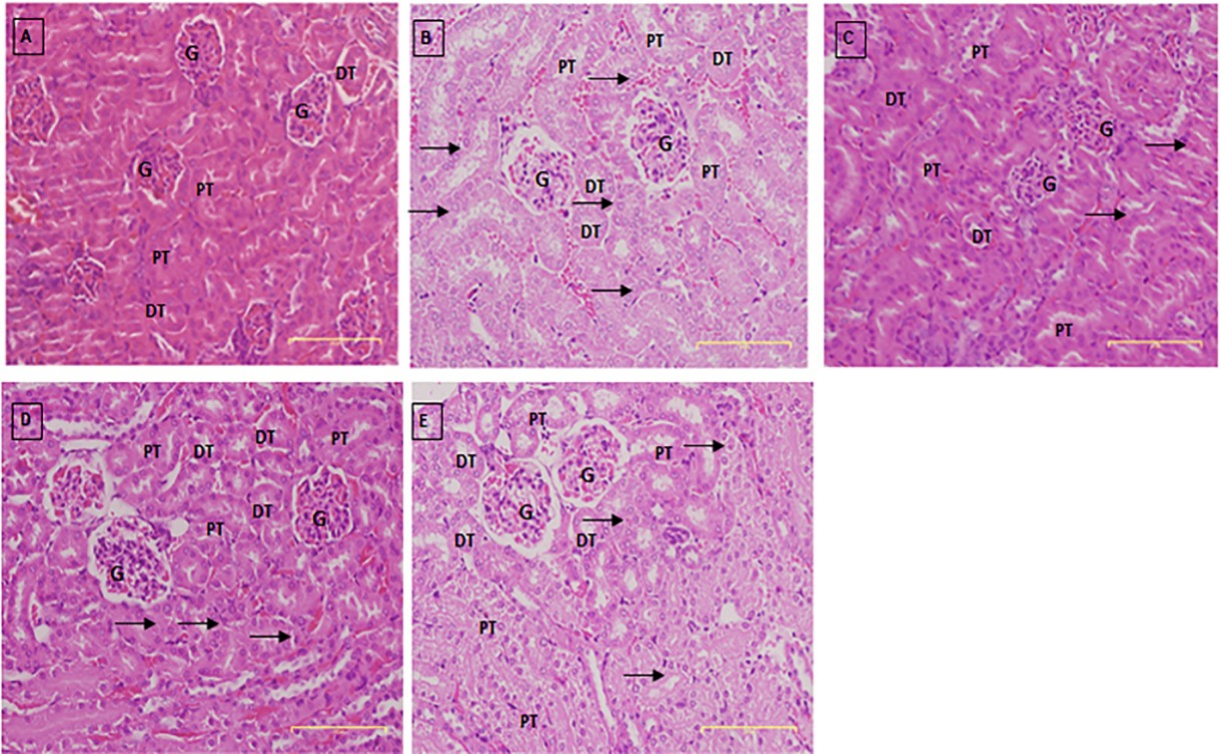
Histology of the kidney after H & E staining in normal mice 4 weeks post drug treatment. Representative sections of the kidney from mice in the A) normal group, B) induced/saline group, C) induced/DOX group, D) induced/PCA-ZnAl group and E) induced/ZnAl-LDH group. The glomerulus and associated tubules showed pronounced changes in B, D and E. Pathological alterations such as dilatation of the glomerulus (G), collecting tubule (PT and DT) and karyomegalic nuclei were evident (arrow). PCA-ZnAl-treated mice (D) were seen to have some diffuse dilated distal tubules (DT), and the ZnAl nanoparticle-treated mice (E) presented more focal dilatation of the tubules (DT). Magnification, 100×.

In the normal group, regular and compacted glomeruli, in addition to numerous tubules, were observed (Fig 10A). In the DEN/PB-induced treated with the saline group, dilatation of the collecting tube, cellular swelling, and karyomegalic nuclei were observed. Changes in renal glomeruli and associated tubules were more pronounced in all groups induced with DEN/PB (Fig 10B–10E). In these three groups, tubular dilatation and inflammatory cell infiltration were prominent. The induced/PCA-ZnAl mice showed some improvement, showing less renal tubular dilation and minimal inflammatory cell infiltration into the glomerulus (Fig 10D). The numbers of dilated distal tubules and swollen, enlarged, and fragmented glomeruli were increased in both PCA-ZnAl and ZnAl-LDH mice (Fig 10D and 10E). However, the dilated distal tubules appeared diffuse in the PCA-ZnAl group, while the swellings were more focal in the ZnAl-LDH group. Some of the proximal tubules in the ZnAl-LDH group lost their lumens due to the swelling of the tubes.

### Biodistribution of LDH nanocarriers in the HCC mouse model

**Fig 11:**
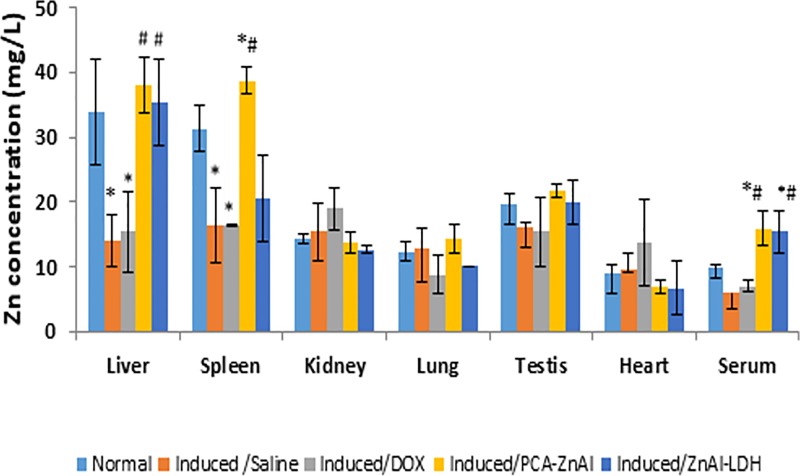
Level of Zn in the tissues and blood of mice after 28 days of drug treatment. Zinc distribution in normal mice and DEN/PB-induced mice treated with saline, DOX, PCA-ZnAl, and ZnAl-LDH. All the data are expressed as means ± SD, n = 8. *p< 0.05 indicates a statistically significant difference compared with the normal group. #*p*<0.05 indicates a statistically significant difference compared with the induced/saline group using one-way ANOVA.

Zinc distribution in the normal and DEN/PB-induced mice was carried out to verify whether the nanoparticles were decomposed, eliminated or accumulated following 28 days of administration. Therefore, the level of Zn, which is a main component of LDH, was analyzed using the atomic absorption spectroscopy (AAS) technique. The results of Zn distribution are depicted in Fig 11. The levels of Zn in the organ and serum of normal mice were 33.79±8.09, 31.32±3.56, 14.22±0.77, 12.53±1.50, 19.67±1.61, 8.92±1.48, and 9.76±0.51 mg/L in the liver, spleen, kidney, lung, heart, testis, and serum, respectively.

The DEN/PB-induced mice treated with saline showed distinct changes in the level of Zn in the liver, spleen, and serum. The Zn concentrations in the liver and spleen of DEN/PB-induced mice treated with saline or DOX were significantly lowered than that of the normal control. A significant increase in the Zn level was observed in the PCA-ZnAl- and ZnAl-LDH-treated groups compared with that in the saline- or DOX-treated group. The Zn level in the spleen of PCA-ZnAl-treated mice was significantly higher than that in saline-treated mice. Furthermore, the level of Zn in the serum of HCC mice treated with PCA-ZnAl and ZnAl-LDH were significantly higher than that in normal or induced/saline control mice. The levels of Zn in the testis, lung, heart, and kidney were not significantly different between the treated groups and normal or untreated control group.

## Discussion

The observations of the body weight, liver features and tumor biomarkers in animals are three initial indicators in assessing the pathological condition of the liver. In this project, DEN/PB-induced HCC mice treated with saline showed weight loss, which corroborated previous findings [21,22]. They stated that the cancer-induced model exhibited reduced weight gain compared with the normal animal [21, 22]. After cancer induction with DEN/PB, the mice developed benign hepatomas, similar to neoplastic nodules that were thought to be the possible precursors of hepatic cancer in experimental animals and humans [23,24]. Livers obtained from DEN/PB-induced mice also showed a significant increase in weight compared with that in the normal group and can be explained by the effect of PB on the liver. The administration of PB is associated with initial transient hyperplasia in rodents as an early event after treatment, and a substantial proliferation of smooth endoplasmic reticulum caused hepatocellular hypertrophy that contributes to the liver weight increment [25]. However, treatment using PCA-ZnAl reduced liver weight, an indication of liver improvement. This observation was in agreement with that in previous studies [11,26]. These results were further confirmed by the histopathological results after H&E staining.

Histopathological examination of liver sections from DEN/PB-induced HCC treated with PCA ZnAl elicited a maximum therapeutic effect against DEN/PB-induced hepatocarcinogenesis. In this group, liver cells were found to contain compact cytoplasmic material with only clear cell foci. The nucleus to cytoplasm ratio of the PCA-ZnAl-treated group was decreased considerably compared with that of the saline-treated group. The configuration of sinusoids appeared normal with normal Kupffer cells. The size of the nuclei resembled that of normal cells, and much fewer binucleated cells were observed. A moderate improvement in the hepatic histological picture was observed in the PCA ZnAl-treated group compared with that in the saline-treated group. However, in the saline-treated group, substantial irregularity of the nucleus shape and extensive vacuolated cytoplasm with a high nucleus to cytoplasm ratio can be observed. The liver sections from these groups also presented increased and greatly dilated sinusoids, with hyperplastic Kupffer cells that represent the cytological make-up of the hepatocytes in liver cancer, findings that are comparable to those reported previously [27]. The ability of PCA-ZnAl to induce the overall improvement in the hepatic histoarchitecture emphasizes the therapeutic potential of PCA-ZnAl. Hence, the present study suggests the potential of PCA-ZnAl in mitigating the hepatic toxicity induced by DEN/PB. Consistent with these observations, it has been previously demonstrated that PCA can ameliorate liver cancer induced by the administration of CCl_4_ in Sprague-Dawley rats [28].

Alpha fetoprotein is a useful diagnostic tumor maker of HCC that has been used for tumor staging, diagnosis, monitoring, and even detecting the recurrence of liver tumors [29]. In this study, a significant increase in the level of αFP was observed upon the induction of HCC using DEN and 12 weeks of PB administration. The level of αFP remains elevated in HCC mice treated with saline. However, the level of αFP was significantly reduced after 28 days of treatment with PCA-ZnAl, suggesting the anticancer action of PCA-ZnAl against hepatocellular carcinoma in mice.

Furthermore, a significant increase in the activities of AST, ALT, and ALP was observed in mice induced with DEN/PB and treated with saline. Liver damage caused by DEN/PB results in the deterioration or destruction of the cell membrane, leading to the leakage of transaminases and phosphatase from the liver tissue into the bloodstream because these are primarily localized in the liver [30]. HCC mice treated with DOX and PCA-ZnAl showed a significant reduction in these liver enzymes compared with the untreated control mice. This demonstrates the ability of PCA-ZnAl to repair the liver injury by maintaining the integrity of the cell membrane, thereby mitigating the progression of carcinogenesis.

It is important to point out that the serum bilirubin level is considered a diagnostic marker of hepatic function in health and disease monitoring [31]. Mouse hepatic damage caused by DEN/PB administration resulted in the increase in the conjugated bilirubin level, reflecting a pathological alteration in the biliary flow. However, treatment with PCA-ZnAl ameliorates the effect by decreasing the total bilirubin level, suggesting the therapeutic potential of the PCA-ZnAl nanocomposite in hepatocellular carcinoma mice.

Two serum protein levels, albumin and globulin, are biochemical hallmarks in the diagnostic criteria of the synthetic ability of the liver. In the present study, the administration of DEN/PB altered the level of the serum albumin/globulin (A/G) ratio, which is indicative of severe impairment in the protein biosynthesis of the liver. The variation in the total serum protein content observed in this study reaffirms the mutagenic action of DEN and PB [32]. DEN causes the dissociation of polyribosomes from the surface of the endoplasmic reticulum, which plays a vital role in protein biosynthesis. This modification disrupts the protein synthesis in the liver which is similar to a previous study [33] after the administration of DEN/PB. The A/G ratio was reversed to a slightly better level in mice treated with PCA-ZnAl due to the increased serum globulin level compared with that in the saline-treated group.

One of the manifestations of liver cancer is associated with hypogonadism and signs of feminization, irrespective of direct effects of testicular atrophy and a low testosterone level. In this study, cryptorchidism syndrome—notably, an undescended testis—was observed in DEN/PB-induced mice treated with saline. This is one of the most common congenital abnormalities of male sexual development characterized by failure of the testis to descend into the scrotum due to growth hormone imbalance, leading to increased risk of male infertility, testicular cancer, germ cell loss, and impaired spermatogenesis [34–36]. The testes of mice induced with DEN/PB and treated with saline were found in the abdomen. Notably, among the changes were the loss of an orderly pattern of cells in seminiferous tubules, degeneration of the germinal epithelium, and increase in the width of the interstitial space on histopathological sections of the testis.

However, mice treated with PCA-ZnAl showed normal testicular development, and the testis was located in the sac in the genital region inside the scrotum similar to the normal mice. The positive effects of PCA nanocomposites seen in this study were similar to that previously reported [37]. They stated that the introduction of PCA provided protection and prevented environmental contaminant toxicity against the reproductive system. It also caused significant reversal of testicular damage, increased serum testosterone levels, and increased sperm motility and sperm count [37]. Similarly, the results of histopathological sections of testis in PCA-ZnAl-treated mice showed a regular spectrum of seminiferous tubules with slight reduction in the number of spermatogonial cells in a narrow lumen. This feature is comparable to the normal testicular architecture shown by normal mice. This observation provides evidence that PCA had a protective effect against DEN/PB-induced morphological and histopathological changes of the testis.

In addition to being well-known as a mineral involved in androgen production and spermatogenesis [38], Zn is also a well-established antioxidant and a key element in the synthesized nanocomposite used in this study. Hence, the preservation of the testicular architecture and other positive histological changes seen in mice induced with DEN/PB and treated with ZnAl-LDH were possibly due to the protection of Zn in the reproductive system.

Existing liver cancer and other longstanding liver damage cause patients to develop hepato-renal syndrome, where liver disease patients experience renal dysfunction [39]. Thus, the analysis of urea, electrolyte, and creatinine levels in mice after 4 weeks of treatment with PCA-ZnAl, ZnAl-LDH, DOX, and saline was also investigated. Except for the potassium (K+) level that was elevated in the DEN/PB-induced group compared with the control group, electrolyte and urea tests were within the same range as those in the control group. A higher level of serum K+ indicates leakage of intracellular potassium from the renal tubular epithelium into blood or a decline in K+ elimination via renal excretion [40]. Disruption in the interwoven structure and function of kidneys may lead to either case. However, in this study, no lesion was detected macroscopically on the kidneys.

In all the DEN/PB-induced mice, significant renal tubular abnormalities were observed compared with those in the normal mice. The swelling of the glomeruli, proximal tubule, and Bowman capsules compared with that in the normal control mice suggested the occurrence of toxicity induced by DEN/PB [41]. However, the tubule dilatation in both PCA-ZnAl- and ZnAl-LDH-treated mice observed in this study was moderate compared with that reported previously [41]. This observation may be due to the action of Zn ion in stabilizing the normal function of cell membranes and inhibiting lipid peroxidation by free radicals [42].

The formation and accumulation of reactive oxygen species (ROS) in hepatocytes account for chemically induced hepatocarcinogenesis [43,44]. Under normal conditions, healthy cells should counter ROS production using an antioxidant defense system such as SOD, CAT, and GSH. However, the administration of phenobarbital, a tumor promoter, renders a strong inhibition effect on this antioxidant defense system, similar to that reported previously [45].

The activities of enzymatic antioxidants (SOD and CAT) and nonenzymatic antioxidant (GSH) in the liver sample were significantly decreased in the DEN/PB-induced mice treated with saline, a finding that agrees with that in the earlier mentioned studies. The decrease in the SOD levels may be due to the enzyme utilization to counteract the excess free radicals generated due to DEN/PB administration. CAT protects hepatocytes from highly reactive hydroxyl radicals by decomposing hydrogen peroxide. This action is thought to be the first line of defense against oxidative damage caused by H_2_O_2_ and other radicals induced by DEN [46,47]. Therefore, the decrease in CAT activity in the DEN/PB-induced group treated with saline may be caused by its high utilization for the removal of H_2_O_2_, which was triggered upon DEN administration. GSH plays an essential role in the well function of cellular biology. During oxidative stress, GSH is converted to GSSG, and GSH exhaustion leads to lipid peroxidation [48,49]. In this manner, GSH was suggested as a marker to evaluate cellular oxidative stress. The depletion of the GSH level in DEN/PB-induced mice might be caused by its excessive usage to prevent the activity of free radicals [50]. The increased degree of lipid peroxidation also correlates with the exhaustion of GSH [51,52].

On the other hand, there was a significant increase in the SOD, CAT, and GSH in the liver of mice administered with PCA-ZnAl compared with DEN/PB-induced mice treated with saline. This increase is due to the ability of PCA-ZnAl to offer protection against oxidative stress. Thus, PCA-ZnAl was proven to have an ameliorative effect on the damage caused by DEN/PB in the liver. The group treated with DEN/PB showed the greatest increase in the levels of TBARS compared with the normal group. This increase in the levels of TBARS indicates enhanced lipid peroxidation leading to tissue injury and failure of the antioxidant defense mechanism to prevent the formation of excess free radicals [51].

The protective ability of PCA, in most cases, may be associated with endogenous antioxidant defense that might be responsible for the antioxidant activity of PCA in biological systems. This might be the result of GSH sparing due to the antioxidant activity of PCA such as substitution for GSH in scavenging radicals [53]. However, it might also be related to the ability of PCA to strengthen the activity of the entire GSH cycle by improving the efficiency of the GSH-related system. It has been shown that PCA can directly activate mRNA transcription and the activity of antioxidant/detoxifying enzymes such as glutathione peroxidase (GPx) and glutathione reductase (GR) in murine macrophages [54]. Therefore, an increase in the level or activity of these antioxidant agents may protect the mouse liver against ROS and nucleophilic ions produced by DEN and PB metabolism [55]. Due to this observation, it can be suggested that the elevation of GSH might be one of the important mechanisms for PCA-ZnAl against DEN/PB-induced hepatocarcinoma in mice.

Surprisingly, ZnAl-LDH appears to significantly increase the SOD and CAT activities and GSH level in mice. Zn is a component of 1,000 proteins, including zinc superoxide dismutase (CuZnSOD). Moreover, zinc atoms are part of zinc finger proteins responsible for the DNA binding activity of transcription factors that induce the expression of catalase [56]. It can be hypothesized that treatment with ZnAl-LDH might have increased the antioxidant defense mechanism against offensive oxidative inducers.

To further understand the effect of nanocomposites on the other organs, we evaluated the histoarchitecture of the lung sections. It is well established that pulmonary damage due to the toxicity of DEN/PB is usually nonspecific, and the damage may be permanent unless measures are instituted [57]. Changes in the lung architecture and lymphatic infiltration were prominently observed upon DEN/PB administration [58]. The lung is packed with inflammatory infiltrates, thereby inducing the inflammatory environment and further exacerbating pulmonary damage. Our results consistently indicated that mice induced with DEN/PB and treated with saline showed increased interstitial tissue replacing the normal capillaries, alveoli, and intact interstitium. However, these pathological changes were significantly reduced after repeated administration of PCA-ZnAl in our study.

Furthermore, *in vivo* biocompatibility and the therapeutic potential of LDH nanocomposites have been investigated by several assays, mainly through the biodistribution and accumulation of the nanocarriers in tissues [59, 60]. As drug diffuses from the plasma to the organs in the whole body, the remnant of LDH nanocomposites in each organ in the *in vivo* experimental animal can be assessed. This assay was carried out to verify whether nanoparticles, as drug delivery systems, are easily decomposed and eliminated from the organ after delivering the drug molecules to specific tissues.

Thus, Zn, a main component of LDH, was analyzed 28 days post injection in DEN/PB mice. No significant accumulation of Zn was found in the organs, except for the liver and spleen, after treatment with PCA-ZnAl and ZnAl-LDH compared with that in the saline-treated group. The accumulation of nanoparticles was more abundant in the liver and spleen in the nanoparticles group, suggesting that nanocomposites are more easily taken up and accumulated in those organs. The predominant accumulation of LDH nanoparticles in the liver may be related to the high number of macrophages in the liver, which contains approximately 80–90% of all macrophages of the body [61]. This preferential in vivo biodistribution of LDH with the size of 100–200 nm has added to the merits in developing organ-specific drug delivery potential in cancer therapy, especially liver cancer.

For over half a century, a few reported population studies have consistently demonstrated a marked decrease of approximately 55–75% in zinc levels in HCC tissue compared with that in normal liver tissue [62–65]. The zinc concentration in the liver of DEN/PB-induced HCC mice treated with saline was significantly less than that in the liver of normal mice. The decreased Zn level in liver disease was attributed to the downregulation of ZIP14, a transporter that moves Zn from the extracellular space into the cellular cytoplasm of hepatoma cells. Although Zn has been proposed to have anticancer properties in multiple systems, it was suggested that the intracellular levels of zinc are downregulated in HCC to suppress its ability to inhibit cancer formation [66].

A low Zn serum status is remarkably prevalent in advanced liver disease [67]. Here, the induction of HCC using DEN/PB also leads to a decrease in the Zn level in mouse serum compared with that in the normal group. Additionally, it was suggested that various factors, including increased gut permeability, diminished intestinal Zn absorption, increased gastrointestinal and urinary excretion, malnutrition, and diminished hepatic Zn, are confounding factors for the low Zn level in the blood [68].

However, treating cancerous mice using the nanocomposite PCA-ZnAl and its nanocarrier increased the zinc level in the liver, spleen, and serum of the DEN/PB-induced mice. An increased concentration of serum zinc compared with the normal value was reported in liver disease patients during 10 and 60 days of zinc administration as a supplement [69]. The role of Zn is remarkable, and this study supported the great potential of Zn supplementation via nanocarriers as an adjuvant in anticancer therapy. We recognize some limitations of this present study. The selection of only a few animals for confirmation of HCC could have introduced selection bias; however, we expect a low risk of selection bias since the animals were randomly selected. Another limitation is that in addition to serum AFP, other HCC markers such as glypican-3, gamma-glutamyl transferase and AFP mRNA expression are important markers for the assessment of HCC, which were not included in the present study. Lastly, the Zn used in the formulation of nanoparticles was not labeled and free Zn in circulation might have influenced the result of Zn level. However, this may not cause a significant impact on the result since there was a normal control group.

## Conclusions

The administration of a single dose of DEN in mice led to the formation of hepatocellular carcinoma, causing significant damage to the lungs, kidney and testis, with the animals presenting with altered liver function parameters and elevation of the tumor marker alpha fetoprotein. However, treatment with PCA-ZnAl nanocomposites and doxorubicin led to remarkable improvement with reversal of the tumor marker expression, slight gain in weight, and reversal of both the lung and kidney structures similar to normal control. Animals treated with synthesized nanocomposites containing PCA showed better or similar improvement in all the listed parameters compared to DOX and plain nanocomposites after 4 weeks of treatment. The pharmacological benefit of natural products, such as PCA, and improved sustained delivery potential of nanocomposites in the treatment of cancers should be studied further to achieve the desired goal in cancer management.

## Supporting information

S1 Checklist (DOCX)

S1 File (XLSX)

S2 File (XLSX)

S3 File (XLSX)

S4 File (XLSX)

S5 File (XLSX)

S6 File (XLS)

S7 File (XLSX)
