# Supplementary material for: Effect of protocatechuic acid-layered double hydroxide nanoparticles on diethylnitrosamine/phenobarbital-induced hepatocellular carcinoma in mice
Source: PLoS One. 2019 May 29;14(5):e0217009. doi: 10.1371/journal.pone.0217009 (PMC6541272; doi:10.1371/journal.pone.0217009)

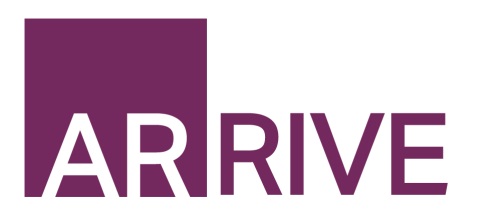


The ARRIVE Guidelines Checklist

Animal Research: Reporting In Vivo Experiments

Carol Kilkenny^1^, William J Browne^2^, Innes C Cuthill^3^, Michael Emerson^4^ and Douglas G Altman^5^

*^1^The National Centre for the Replacement, Refinement and Reduction of Animals in Research, London, UK, ^2^School of Veterinary Science, University of Bristol, Bristol, UK, ^3^School of Biological Sciences, University of Bristol, Bristol, UK, ^4^National Heart and Lung Institute, Imperial College London, UK, ^5^Centre for Statistics in Medicine, University of Oxford, Oxford, UK.*

|  | | ITEM | RECOMMENDATION | Section/ Paragraph |
| --- | --- | --- | --- | --- |
| 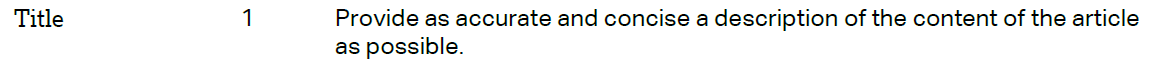 | | | Title |  |
| 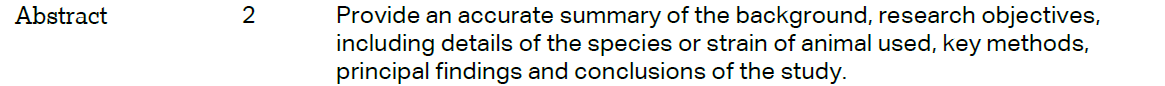 | | | Abstract |  |
| INTRODUCTION | | |  |  |
| 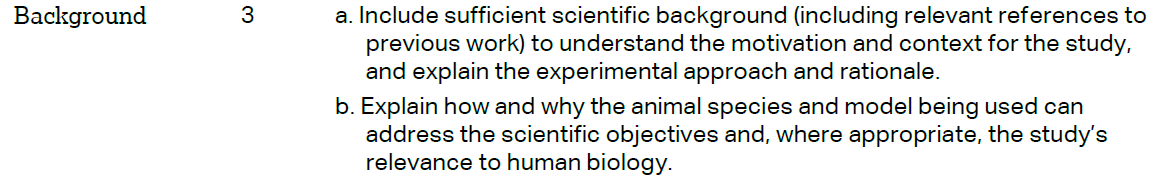 | | | Paragraph 1-4  Paragraph 5-6 |  |
| 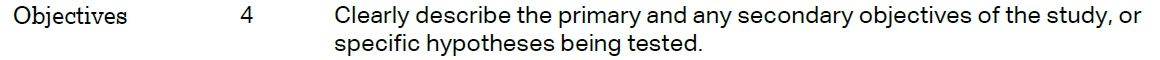 | | | Paragraph 6 |  |
| METHODS | | |  |  |
| 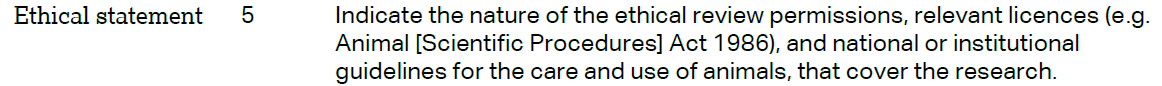 | | | Animal preparation- last sentence |  |
| 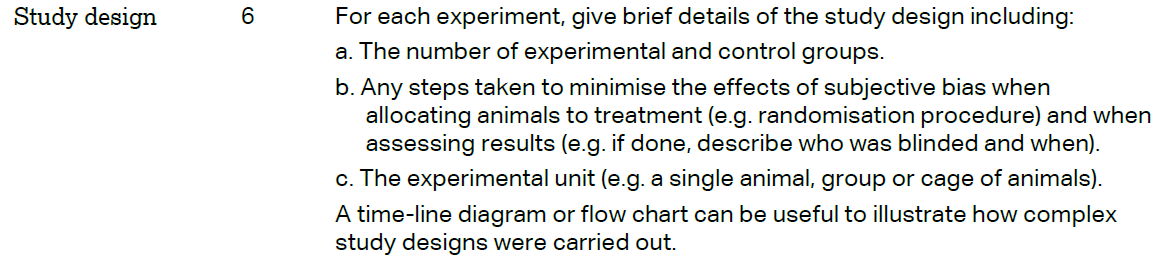 | | | Induction of HCC  Induction of HCC  Induction of HCC |  |
| 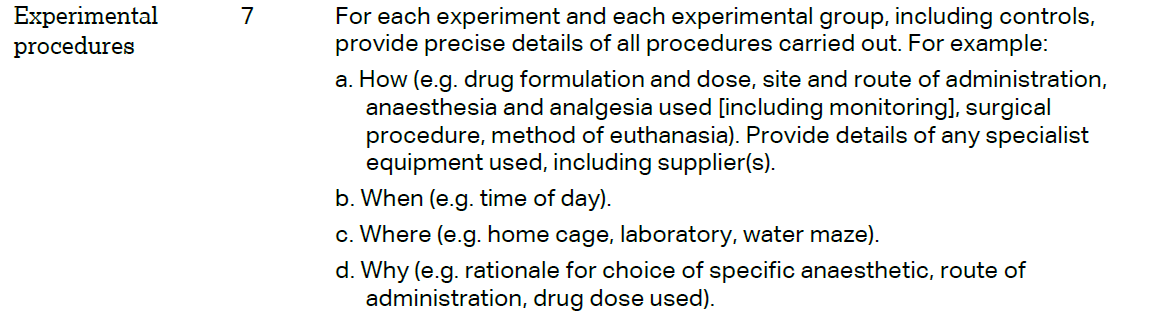 | | | Animal prep, induction of HCC, characterization of nanoparticles  Induction of HCC    ,, |  |
| 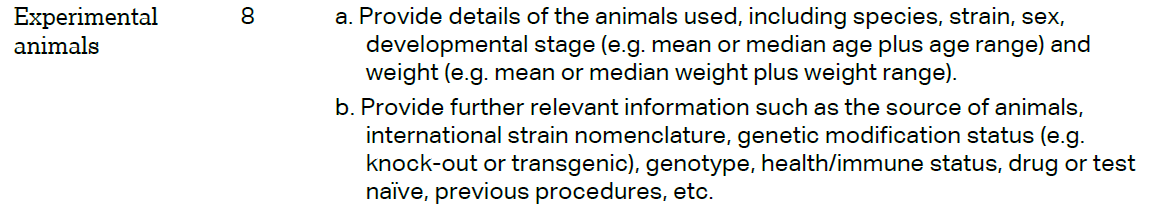 | | | Animal prep, induction of HCC  ,, |  |

The ARRIVE guidelines. Originally published in *PLoS Biology*, June 2010^1^

| 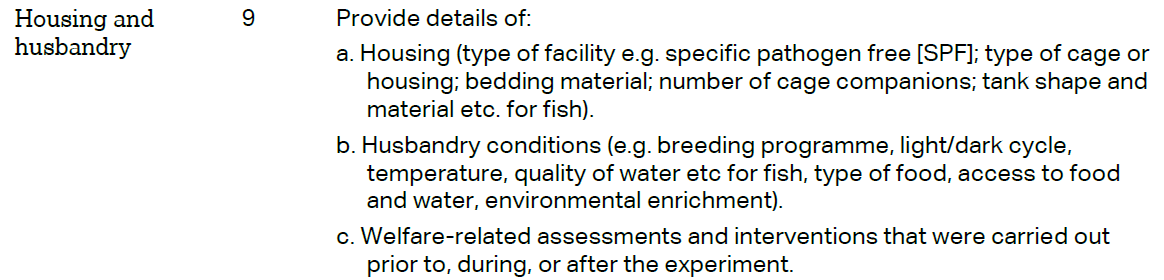 | Animal prep  ,,  Animal prep, induction of HCC | |
| --- | --- | --- |
| 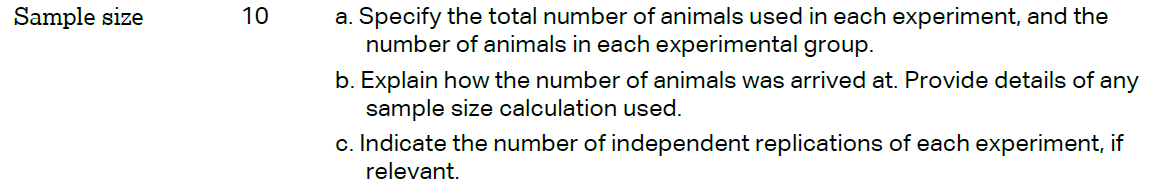 | Animal prep, induction of HCC  Induction of HCC  Induction of HCC, animal treatment | |
| 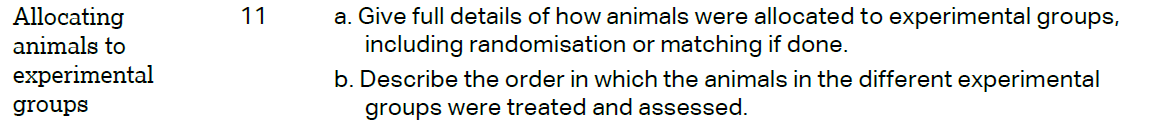 | Induction of HCC  Animal treatment | |
| 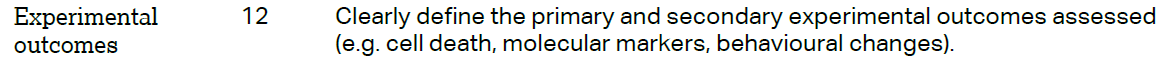 | Confirmation, treatment, sacrifice, histopathology, biochemical analysis, antioxidant status assay, distribution of nanoparticles | |
| 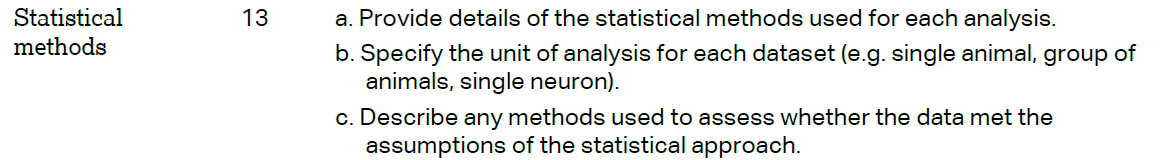 | Statistical analysis  ,, | |
| RESULTS |  | |
| 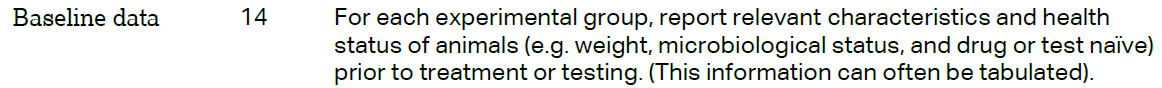 | Induction of HCC | |
| 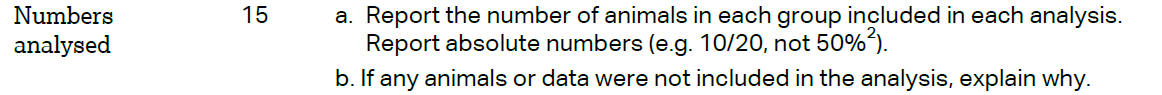 | Results | |
| 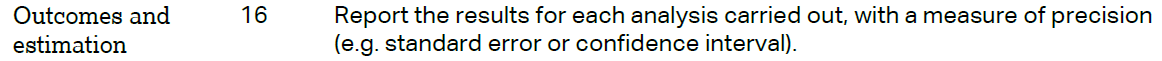 | Statistical analysis, results | |
| 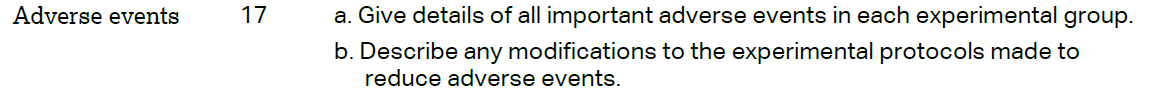 | Induction of HCC | |
| DISCUSSION |  | |
| 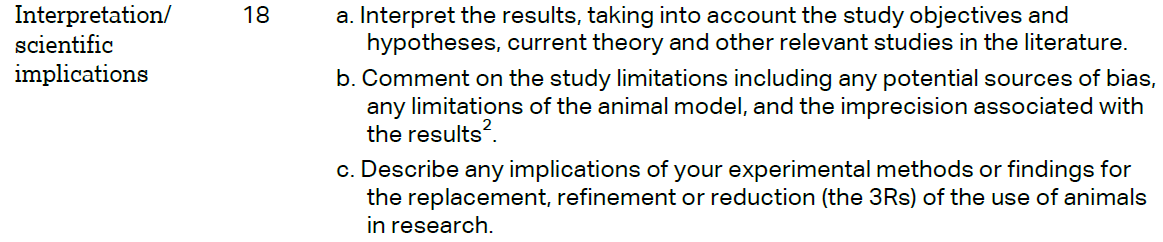 | Throughout  Last paragraph of discussion | |
| 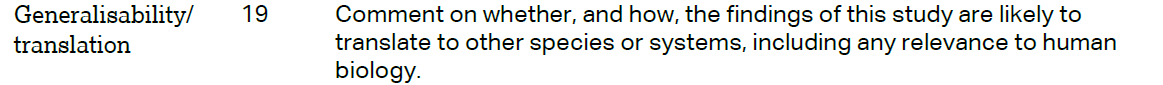 | conclusions | |
| 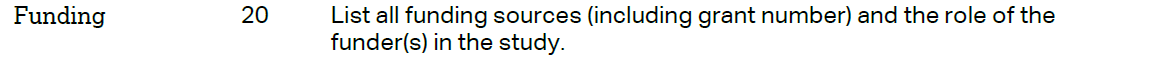 | | Acknowledgements |


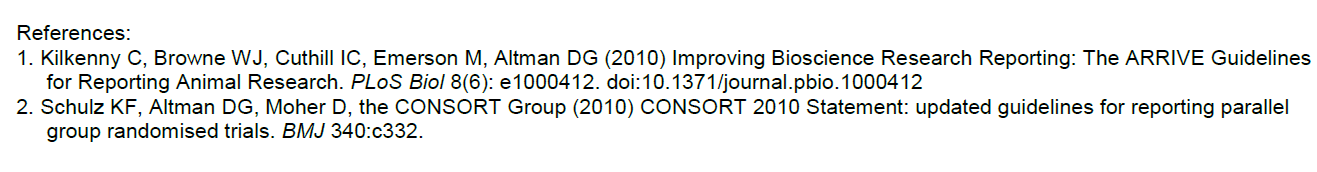

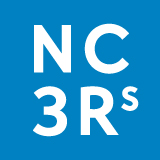

Supplement: S1 Checklist — (DOCX) [file pone.0217009.s001.docx]
